# The Power of Advocacy: Advancing Vision for Everyone to Meet the Sustainable Development Goals

**DOI:** 10.3389/ijph.2022.1604595

**Published:** 2022-07-06

**Authors:** Eva Lazuka-Nicoulaud, Kovin Naidoo, Kristan Gross, Judith Marcano Williams, Andrea Kirsten-Coleman

**Affiliations:** ^1^ Vision Impact Institute, Paris, France; ^2^ OneSight EssilorLuxottica Foundation, Durban, South Africa; ^3^ Vision Impact Institute, Dallas, TX, United States

**Keywords:** public health, Sustainable Development Goals, SDGs, advocacy, eye health, vision care, uncorrected refractive error, URE

## Abstract

Advocacy is instrumental to achieving significant policy change for vision. Global advocacy efforts over the past decades enabled recognition of vision as a major public health, human rights, and development issue. The United Nations General Assembly adopted its first-ever Resolution on vision: “Vision for Everyone—Accelerating Action to Achieve the Sustainable Development Goals (SDGs)” on 23 July 2021. The Resolution sets the target and commits the international community to improve vision for the 1.1 billion people living with preventable vision impairment by 2030. To fulfill their commitments, governments and international institutions must act now. Advocacy remains instrumental to mobilize funding and empower governments and stakeholders to include eye health in their implementation agenda. In this paper, we discuss the pivotal role advocacy plays in advancing vision for everyone now and in the post-COVID-19 era. We explore the link between improved eye health and the advancement of SDGs and define the framework and key pillars of advocacy to scaling-up success by 2030.

## Introduction

### The Growing Challenges for Global Advocacy

The global need for advocacy and action grows with the increasing unmet needs in eye care. According to The United Nations (UN) Resolution on vision [A/RES/75/310], global eye care needs will increase substantially, with half of the global population (4.8 billion) expected to be living with a vision impairment by 2050. The aging society, population growth and environmental factors make the challenge ever-growing. The Lancet Global Health Commission reminds that 2.2 billion people around the world live with vision impairment or blindness and 1.1 billion people have vision impairment that could have been prevented or is yet to be addressed [1]. Without action, it is projected that this will rise to 1.8 billion people by 2050. Moreover, 90% of preventable vision impairment occurs in low- and middle-income countries, and 55% of people with vision loss are women and girls. Vision impairment is estimated to cost the global economy US$411 billion in productivity each year. The UN Resolution creates new expectations for international financial institutions and donors to provide targeted finances. Addressing these unmet needs requires a continuum of evidence-driven advocacy and concerted actions.

At the end of 2019, two landmark reports drew attention to global data and effective strategies for eye care. The World Health Organization (WHO) World report on vision evidenced the magnitude of eye conditions globally and proposed an “integrated people-centered eye care (IPCEC)” approach to strengthen health systems and service delivery based on people’s individual needs [2]. A report by Essilor, Eliminating Poor Vision in a Generation, defined the global scale of uncorrected refractive errors (URE) and outlined a roadmap to eliminate this issue by 2050 [3]. While the WHO focused on ending avoidable vision impairment (visual acuity cut-off 6/12) and Essilor specifically targeted URE (visual acuity cut-off 6/9), both reports drew similar conclusions. Both underscored the pivotal role of awareness and advocacy and recommended actions through a full-systems approach, involving governments, multilateral organizations, healthcare organizations, non-governmental organizations (NGOs) and the private sector.

### What Is Advocacy?

The word *advocacy* comes from the Latin *advocāre,* meaning “to call for.” Advocacy is a process of influencing policymakers, stakeholders and targeted audiences, to address a specific issue and attain a particular outcome. Advocacy is not a scientific art. However, it requires strong evidence, data and facts supported by research. High quality information makes advocacy credible, trusted, and impactful when it comes to:

• Raising awareness and educating the audience,
• Engaging and mobilizing stakeholders showing the direction,
• Empowering legislators and decision-makers,
• Ultimately, changing behaviours and policies

To be effective, advocacy requires clarity of purpose, rigorous planning and subsequent monitoring, involving structured approaches such as intervention mapping [4]. Finally, to be successful, advocacy must be supported by coalitions and partnerships and aimed at a wide audience. We define the six-step framework for successful advocacy as follows:

(1) Identify the issue. What is the problem that requires a solution? Analyze the issue (its nature, extent, causes, short-term effects, and long-term impacts) and the socio-political context. Gather evidence about the problem. Define the position and agree on recommendations to solve the problem.
(2) Decide on a goal. What is the ultimate result to be achieved? A goal describes the impact on people’s lives or the world in which they live. A goal is a longer-term measure (ultimate outcome) than an objective (output from an intervention).
(3) Define objectives. Translate the goal into SMART—specific, measurable, achievable, relevant, and timely objectives. The objectives can be set at the level of people (knowledge, skills, behavior, and attitude) or institutions (policy, practice). Each advocacy objective can be local, national or regional. Each objective should focus on specific actions (educating, engaging, mobilizing) to achieve a particular change.
(4) Define strategy. For each objective, define the action plan;
  (4a) identify target audiences (public, policymakers, decision-makers, and people who can influence them),
  (4b) develop evidence-based messaging tailored to each audience,
  (4c) build partnerships and alliances with other advocacy groups, organizations, or individuals, who can support the plan,
  (4d) set activities (intervention scope, channels of communication, timeline).
(5) Implement. Once the action plan is approved (budget, resources, risk assessment, M&E-monitoring and evaluation plan), start campaigning (lobbying, influencing, mobilizing public and media) and remain flexible along the process.
(6) Monitor success. For any project, the following steps are imperative: monitor campaign progress and evaluate results against the plan (activities, indicators, audience reactions), revise, adjust, end the project and report outcomes.

### Defining Advocacy for Vision

“Advocating for eye health means working to change the policies and practices of those institutions, and the attitudes and behaviours of those individuals, whose actions affect the elimination of avoidable blindness” [5]. Advocating for good vision aims, more broadly, at changes that can enable the elimination of uncorrected refractive error. However, an internationally standardized definition of advocacy for vision does not exist, and different organizations and advocacy groups can and do use descriptions specific to their own activities.

### Two Strategic Approaches can Define Advocacy Action Plans

• Structured advocacy programs—engaging and mobilizing governments, non-profits, the private sector, and community members to discuss eye health problems, build partnerships to influence decision-makers, and share efforts to solve targeted issues.
• Communication advocacy—raising awareness and educating around vision issues while using media to reach broader audiences and gain more attention from decision-makers (media advocacy) or any other material and mechanism, such as guest presentation, public event (communication advocacy).

Advocacy can include lobbying (talking directly to decision-makers and those who influence them) and campaigning, which aims to mobilize public and media support for a specific vision issue. Advocacy starts with evidence-based information and, when needed, can involve communication, publicity, education and fundraising practices.

### Defining Key Advocacy Pillars

There are numerous reasons for advocating for eye health and vision care:

• Vision is a global health concern, as well as a human right and economic development issue
• Good eye health is essential for achieving the UN Sustainable Development Goals (SDGs)
• Uncorrected refractive error is the world’s largest avoidable disability
• Poor vision affects education and the ability to reach a child’s full potential
• Vision is vital for safe mobility on the road
• Myopia (near-sightedness) epidemic has become a major public health issue
• COVID-19 pandemic exacerbated visual needs (including myopia risks) across the world
• Poor vision implies potential productivity loss
• Vision affects gender equity
• Eye protection is as important as eye correction for preventing avoidable vision loss
• Cost-effective solutions to the problem already exist. Glasses and other forms of optical correction are a powerful instrument for social and economic development.

Targeting advocacy pillars helps to draw attention to patients’ needs, raise awareness of specific vision problems and their impact; gain access to the human, material, and financial resources; influence policy; and inform eye health practitioners of best practices.

## Discussion

### Everyone can Be an Effective Advocate

Everyone involved in vision care, education, health and economic development can be a successful advocate. This should challenge everyone to view advocacy as part of daily work and strategy. More people, including those outside the ophthalmic industry, must be involved in advocacy and in promoting healthy vision.

Advocacy should not only be part of eye care strategies in practice but also academic culture and education curriculum. Students and academic researchers have an important role to play in advancing health and science policy [6]. Graduate students should get involved in advocacy early enough to become leaders in their field of passion [7]. When everyone is driving for change at the individual level, advocacy’s power lies in collective effort. As the COVID-19 pandemic strongly affected advocacy work in all regions around the world, creating partnerships and coalitions became even more important in the context of this health crisis.

### Advocacy Lessons Learned From the Past

Advocacy has been an important component of global health promotion for the past decades. It has been instrumental in building evidence, strengthening national programs, and supporting the development of national eye care plans.

The WHO World report on vision highlighted substantial progress made thanks to “concerted global advocacy” and actions during the past 30 years. “In 1999, the global initiative for the elimination of avoidable blindness, Vision 2020: The Right to Sight,” strengthened global advocacy initiatives, reinforced national programs for the prevention of blindness and the development of domestic eye care plans. This initiative has been pivotal in achieving unified and coordinated advocacy for key priorities in eye care at a global, regional and national level. “Four World Health Assembly (WHA) resolutions adopted in 2003 (WHA56.26), 2006 (WHA59.25), 2009 (WHA62.1) and 2013 (WHA66.11) have maintained this momentum” and made considerable progress in implementing WHO action plans toward universal eye health and access to comprehensive eye care services. At the same time, the number of population-based surveys undertaken to measure vision impairment and blindness around the world has increased [8] and knowledge generated through this research has been pivotal to advocacy informing public health strategies. In addition, advocacy efforts have been essential for expanding eye care indicators within primary care.

Aligning eye care programming to the broad health and development agendas was also a crucial advocacy effort. A broad all-encompassing coalition of all stakeholders provided a solid platform for effective and persistent advocacy for government support of eye care. The identified common factors for successful advocacy at the national level included an effective gathering of data, using evidence, and lobbying for political commitment [9].

Lessons learned from past successes show that advocacy is crucial to meeting several challenges. The age-standardized global prevalence of blindness fell by 28.5% between 1990 and 2020, and the prevalence of major infectious causes of blindness (onchocerciasis and trachoma) has declined substantially.

### Advocacy to Address Growing Challenges

The global need for eye care, increasing dramatically due to demographic and lifestyle changes, will have a direct impact on health systems. Despite recent scientific and technological progress, significant challenges lie ahead. Advocacy can—and must—address these challenges:

• Uncorrected refractive error—with one in three people affected globally, URE is the world’s largest unaddressed disability on the global health agenda [10]. URE drives children into poverty by limiting their opportunities for education and employment, and can seriously affect their quality of life and academic performance. Advocating for a world free from URE would require creating public awareness to drive consumer demand for vision correction, as well as increasing public sector investment in eye care resources. Both within and outside public health, coalitions will be effective in catalysing efforts and campaigning for change. Providing policymakers with conclusive evidence that indicates the detrimental effects of URE on productivity loss and individuals is needed to mobilize and scale up efforts.
• Myopia burden—the estimates show that the number of people with myopia and high myopia will increase sharply in decades to come. Without action, more than 50 percent of the world’s population will suffer from myopia by 2050 [11]. Advocacy can be the change-catalyst for myopia’s trajectory. Controlling the myopia epidemic will require cross-sectoral efforts addressing the issue from early stages. Modifying lifestyle factors in young children is likely to prevent or delay myopia onset. Another benefit would be a reduction of eye health problems as a complication of myopia into adulthood. Advocacy is needed to facilitate support from families, governments, health and educational bodies to influence child behaviors. Regarding policy change, eye care professionals can influence health systems and ensure the availability of eye exams for all children entering schools and free glasses to those who cannot afford them.
• Presbyopia and healthy aging—if not corrected or under-corrected, presbyopia affects the quality of life of individuals and has negative implications towards employment, productivity, and economic development. Age-related vision disorders should not be accepted as normal. This requires not only education and awareness raising, but also advocacy building to enable health policy focus on near-vision correction.
• Workforce and access—Promotion of education standards for optometrists [12], optometric technicians and optical technicians, and the acceptance of optometry as a profession remain important advocacy issues going forward in many countries. However, advocating for the optometry profession alone is not enough. Advocacy must support a team approach to addressing a crisis that needs collective action to achieve the scale needed for sustainable access to eye care.
• Regional gaps—the magnitude of vision loss, lack of eye health promotion, weak eye health systems, poor quality of eye care services, workforce shortages, fragmented services, lack of coordinated implementation, research gaps and paucity of data are the challenges affecting especially low- and middle-income countries [13]. Evidence-based advocacy must support governments to reinforce their national health plans to achieve universal eye health coverage [14].

### Advocacy to Alleviate the Cost of Poor Vision

In 2021, The Lancet Global Health Commission suggested that annual global economic productivity loss from vision impairment was approximately US$ 411 billion purchasing power parity. Previous research investigated the economic impact of poor vision at the regional and global levels and provided alarming figures:

• The economic burden of uncorrected myopia in the regions of South and East Asia was reported to be “more than twice that of other regions and equivalent to more than 1% of gross domestic product”,
• The annual global “costs of productivity losses associated with vision impairment from uncorrected myopia and presbyopia alone were estimated to be US$ 244 billion and US$ 25.4 billion, respectively”,
• A study among nine countries estimated that “the annual cost of moderate to severe vision impairment ranged from US$ 0.1 billion in Honduras to as high as US$ 16.5 billion in the United States of America”.

**FIGURE 1:**
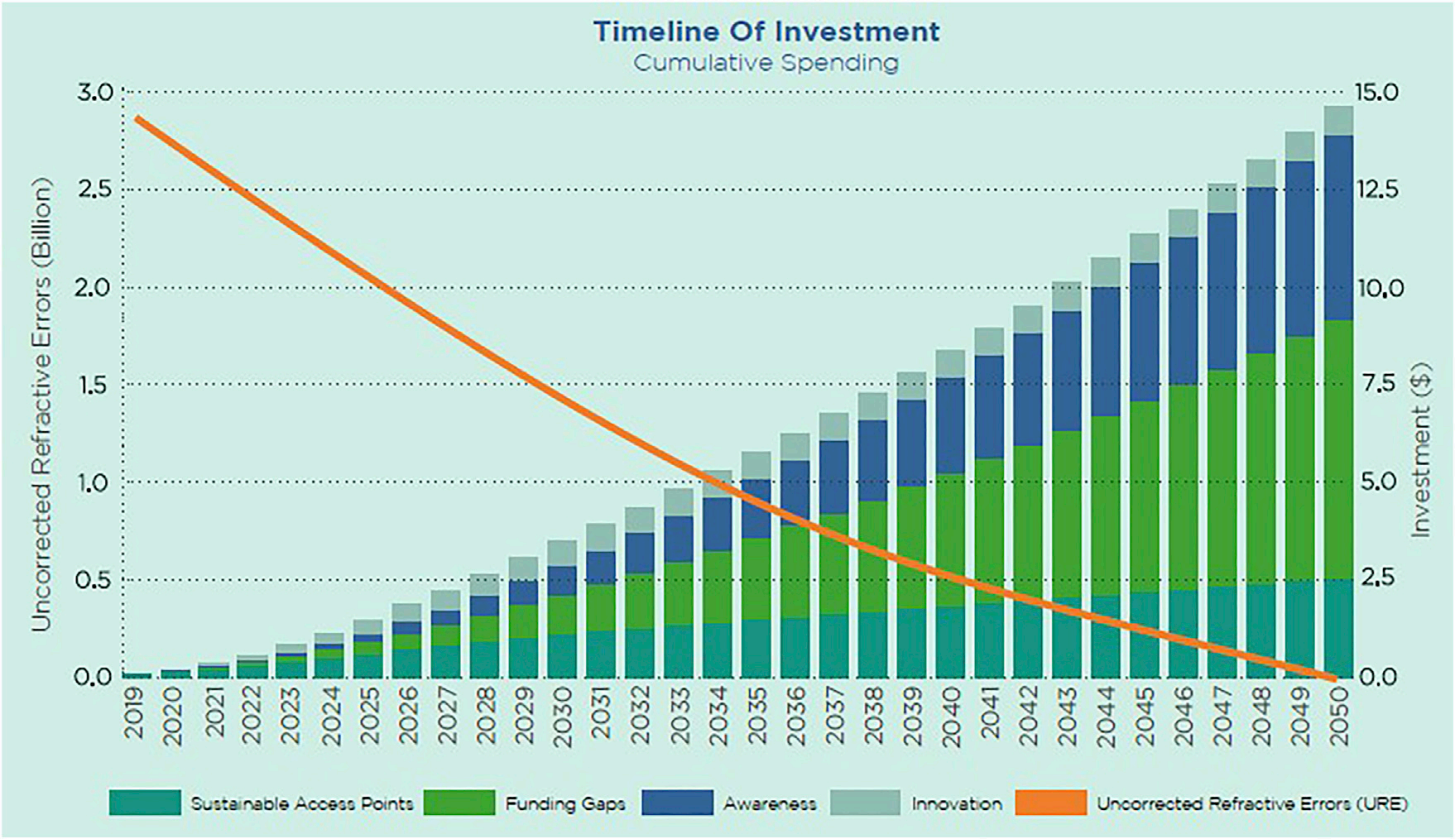
Chart representing the investments needed to create a world free from uncorrected refractive error according to the Essilor’s report “Eliminating poor vision in a generation. What will it take to eliminate uncorrected refractive errors by 2050?” (Global, 2019).

The cost of poor vision dramatically affects the economy, and investments are the solution. The report by Essilor estimates that a total investment of US$ 14 billion over the next 30 years can create a world free from URE by 2050 (Figure 1). The investments include:

• US$ 2.4 billion to create one million new sustainable access points which will equip 90% of the population in need,
• US$ 0.7 billion for innovation to accelerate the affordability of cost-to-serve and cost of products,
• US$ 4.5 billion to increase awareness of poor vision and its socio-economic impact at an individual and societal level,
• US$ 6.2 billion to fill funding gaps across affordability and access for people unable to pay for services.

Advocacy must accelerate funding commitments to address significant gaps and support access to eye health services, primarily within the national health care systems, but also in wider settings including education, industry, and businesses.

### Advocacy: A Change Catalyst for Vision 2030

Advocacy has power to galvanize action, drive change and ensure eyesight is a global political, health and development priority. Resulting from incessant advocacy efforts, the UN Resolution on vision calls for action by governments, civil society, private sector, and the UN institutions to make eye care part of their national agenda to achieving the SDGs by 2030.

The first step is to ensure governments translate their commitment into effective actions, while adopting mechanisms to monitor global progress. One of the mechanisms considered by the UN Inter-Agency Expert Group consists of including two new targets on eye health (effective coverage of refractive error and cataract surgery) in the global indicator framework for the SDGs. The other consists of having countries report on their progress on eye health as part of their Voluntary National Reviews (VNRs) presented at the High-Level Political Forum, which is the main reporting mechanism for the SDGs.

Advocacy is instrumental to implementing the above framework, encouraging governments, and urging international financial institutions and donors to provide financing, especially to developing countries. Advocacy should also encourage collaboration between private sector and government so entrepreneurship can contribute to national efforts to achieve access at scale.

### The Way Forward—Advocacy Messaging With SDGs at the Core

The UN has set ambitious SDG targets for 2030. The UN Resolution on vision starts with consistent advocacy messaging; underscoring that eye health is integral to advancing sustainable development. Extensive evidence, provided by The Lancet Global Health Commission, shows that improving eye health contributes directly and indirectly to over half of the UN SDGs. The following list summarises the key links between eye health and the SDGs:

• SDG 1: No poverty—Poverty is both cause and consequence of poor vision. 90% of vision loss occurs in low- and middle-income countries, with the poorest population affected the most. Vision impairment costs the global economy US$411 billion in productivity loss every year.
• SDG 2: No hunger—Eye care can increase household income and reduce hunger: 46% of households improved their income following cataract surgery.
• SDG 3: Good Health and wellbeing—Eye health is essential to ensuring good health, mental health and wellbeing. Poor eye health affects autonomy and increases the risk of mortality up to 2.6 times.
• SDG 4: Quality education—Eye health affects school enrolment, learning and educational attainment. 91 million children have a vision impairment but do not have access to eye care services. Glasses can reduce the odds of failing a class by 44%. Children with vision impairment are 2–5 times less likely to be in formal education in low- and middle-income countries.
• SDG 5: Gender equality—Women and girls more likely to have vision issues and experience additional barriers to eye care services—55% of people with vision impairment are women and girls.
• SDG 8: Decent work and economic growth—Good vision improves inclusive economic growth, employment and living standards. Glasses can increase workplace productivity by 22%. Cataract surgery can increase household per capita expenditure by 88%.
• SDG 10: Reduced inequalities—Vision loss is driven by inequality. Women, children, older people, persons with disabilities, indigenous peoples, local communities, refugees and internally displaced persons and migrants are most affected by poor vision. 73% of people with vision loss are over the age of 50.
• SDG 11: Sustainable cities and communities—Eye health is critical to reducing road traffic deaths and injuries. An unoperated cataract can increase the chance of a motor vehicle accident by 2.5 times.
• SDG 13: Climate action—The eye health sector contributes to all greenhouse gas emissions and has other impacts on the environment. Climate change may also affect eye conditions and disrupt eye care delivery.
• SDG 17: Partnership—Partnerships and alliances are essential to scaling-up success for vision for everyone by 2030.

To guide future advocacy strategies, additional research is needed to explore the link between improved eye health and the advancement of societal challenges across all SDGs [15].

### Conclusion

A global health problem at a scale of billions of people requires collaboration and systemic initiatives advancing universal access to eye health by 2030. Countries must ensure full access to eye care services for their populations, making eye health integral to their mandate to achieve the UN SDGs. Encouragingly; more than 90% of people with vision impairment have a preventable or treatable cause with existing highly cost-effective interventions. Investing in universal eye health coverage is a realistic, cost-effective means of unlocking human potential. While the remaining challenges are considerable, solutions exist, including interventions for health promotion, prevention, care and rehabilitation to address the full range of eye care needs. Evidence-based advocacy is essential for making good vision a global priority. From the perspective of the SDGs, advocacy is crucial to create an enabling environment for eye care delivery and support the nations to reach their commitments for 2030.

## Definitions

Eye health—The Lancet Global Health Commission on Global Eye Health defined eye health as the state in which vision, ocular health, and functional ability are maximised, thereby contributing to overall health and wellbeing, social inclusion, and quality of life. Eye health can be considered both a process and an outcome. The Commission defined eye care services as those that contribute to any of vision, ocular health, or functional ability being maximised.

Uncorrected refractive error (URE) refers to refractive errors that could be corrected with glasses or other solutions but have not been. Myopia, hyperopia, astigmatism, and presbyopia are the four most common refractive errors.

## Search Strategy and Selection Criteria

References, peer-reviewed articles and relevant reports for this paper were identified by searches through MEDLINE, PubMed, and visionimpactinstitute.org, using the search terms: “advocacy,” “eye health,” “vision care,” “Sustainable Development Goals,” “SDGs,” “uncorrected refractive error,” and “public health.” Only articles published in English between 2000 and 2021 were included.
